# Possible options for introducing quality aspects in Activity-Based Funding pricing

**DOI:** 10.1186/1472-6963-15-S2-A2

**Published:** 2015-06-15

**Authors:** James Downie

**Affiliations:** 1 Independent Hospital Pricing Authority, Sydney, New South Wales 2000, Australia

## Background

In 2011, all of the Australian governments committed to health care reform in which they agreed to use a nationally consistent “Activity-Based Funding” approach for public hospital services in Australia. This reform sought to increase the transparency of public hospital funding and improve the efficiency of public hospital services. As part of this reform, the Independent Hospital Pricing Authority (NIHA) was created, to determine the National Efficient Price (NEP) for public hospitals services, as well as the classification, costing, and counting standards used in setting the NEP.

One criticism of Activity-Based Funding is that it fosters increases in hospital activity without measuring quality, safety, or outcomes. To address this criticism, in the last two years, the IHPA has begun to develop several options for introducing quality components into the NEP. The work has been carried out in partnership with the Australian Commission on Safety and Quality in Healthcare, and is overseen by a Joint Working Party (JWP) comprised of senior clinicians from across the country. This paper gives an overview of the time-consuming and complex, but ultimately productive, work that has been carried out to date.

## Materials and methods

The JWP conducted a Literature Review about healthcare pricing models. The JWP also commissioned a project to undertake a clinician-led process to identify, specify, and group a national set of high-priority Hospital-Acquired Complications, in order to facilitate routine local reporting and review. The project included three key activities:

1. A review of safety literature and of reports from hospital incident reporting systems;

2. A clinician-led and iterative identification of high-priority complications; and

3. An analysis of identified complications that used hospital inpatient morbidity data to support clinician-identified complications.

Finally, the JWP assessed the impact of removing Hospital-Acquired Complications from DRG assignment. Its analysis of the existing administrative datasets for admitted patients demonstrated that a Hospital-Acquired Complication is reported for about 10% of patients.

## Results

The literature review revealed that a rich literature exists that argues these models should reward quality and safety and many of the arguments in the literature are inherently appealing. While strong in their arguments, however, we found that most of the literature is weak on evidence.

The review project identified 39 high-priority preventable complications, 37 of which can be measured in the existing patient diagnosis datasets using the Condition On-Set Flag. The JWP subsequently launched a six-month proof-of-concept project to test this set of preventable complications’ utility at the hospital level.

The JWP found that a Hospital-Acquired Complication diagnosis costs around A$9,000 per episode, and increases the patient’s length of stay by approximately 5 days. The impact of ignoring these diagnoses in DRG assignment is much smaller, however, with only 3.1% of cases impacted.

## Conclusions

This paper provides a detailed overview of the JWP’s work to date, and highlights future areas for work.

